# Hair Growth Effect of DN106212 in C57BL/6 Mouse and Its Network Pharmacological Mechanism of Action

**DOI:** 10.3390/cimb45060322

**Published:** 2023-06-09

**Authors:** Ji Yun Baek, Byoung Ha Kim, Dong-Wook Kim, Won-Yung Lee, Chang Eop Kim, Hyun-Young Kim, Jaesung Pyo, Eun-Seok Park, Ki Sung Kang

**Affiliations:** 1 College of Korean Medicine, Gachon University, Seongnam 13120, Republic of Korea; wldbsttn@naver.com (J.Y.B.); lwy21@gachon.ac.kr (W.-Y.L.); eopchang@gachon.ac.kr (C.E.K.); 2 School of Pharmacy, Sungkyunkwan University, Suwon 16419, Republic of Korea; mot37@d-nature.co.kr; 3 College of Pharmacy, Wonkwang University, Iksan 54538, Republic of Korea; pharmengin1@wku.ac.kr; 4 Department of Food Science, Gyeongnam National University of Science and Technology, Jinju 52725, Republic of Korea; hyunyoung.kim@gnu.ac.kr; 5 College of Pharmacy, Kyungsung University, Busan 48434, Republic of Korea; jspyo@ks.ac.kr

**Keywords:** *Centipeda minima*, DN106212, hair growth, *Vegfa*, *Igf1*

## Abstract

*Centipeda minima* (CMX) has been widely investigated using network pharmacology and clinical studies for its effects on hair growth via the JAK/STAT signaling pathway. Human hair follicle papilla cells exhibit hair regrowth through the expression of Wnt signaling-related proteins. However, the mechanism of action of CMX in animals has not been elucidated fully. This study examined the effect of induced hair loss and its side-effects on the skin, and observed the mechanism of action of an alcoholic extract of CMX (DN106212) on C57BL/6 mice. Our results showed that DN106212 was more effective in promoting hair growth than dimethyl sulfoxide in the negative control and tofacitinib (TF) in the positive control when mice were treated with DN106212 for 16 days. We confirmed that DN106212 promotes the formation of mature hair follicles through hematoxylin and eosin staining. We also found that the expression of vascular endothelial growth factor (*Vegfa*), insulin-like growth factor 1 (*Igf1*), and transforming growth factor beta 1 (*Tgfb1*) is related to hair growth using PCR. DN106212-treated mice had significantly higher expression of *Vegfa* and *Igf1* than TF-treated ones, and inhibiting the expression of *Tgfb1* had similar effects as TF treatment. In conclusion, we propose that DN106212 increases the expression of hair growth factors, promotes the development of hair follicles, and promotes hair growth. Although additional experiments are needed, DN106212 may serve as an experimental basis for research on natural hair growth-promoting agents.

## 1. Introduction

Hair loss results in increased hair fall and thinning of hair on the scalp due to both environmental and genetic factors [1]. Types of hair loss include male-type, female-type, circular, and telogen hair loss [2]. Androgen, a male hormone, is considered to be an important factor in male pattern baldness, characterized as a widening the forehead in an M-shape and the loss of hair in the parietal part [3]. It is also one of the causes of female pattern hair loss; however, compared to male pattern hair loss, the hairline on the female forehead is well maintained, but the hair becomes thinner overall and the parting area is widened [4]. Alopecia areata is caused by alopecia occurring in one or several parts, and the cause is not clearly specified, but it is known that it is caused by stress or an autoimmune response [5,6]. Although the cause of telogen effluvium cannot be specified, hair falls out due to nutritional deficiencies, childbirth, and external influences [7]. The number of people suffering from hair loss is increasing, largely due to environmental factors and other factors such as diet and stress [8,9,10]. Hair is often used as a medium of expression, which gives it more of an aesthetic than functional value [11]. In healthy people, the hair growth cycle comprises anagen, catagen, and telogen. If the cycle is not properly regulated, telogen continues, resulting in hair loss [12]. Enhanced activity of 5α-reductase activates testosterone by converting it into dihydrotestosterone (DHT) that inhibits cell division, resulting in alopecia [13]. 

*Centipeda minima* (CMX) is distributed in the Republic of Korea, China, and India, where it grows well on roadsides or in wet gardens [14]. In China, it is used to treat chronic rhinitis, headache, and eye diseases [15]. It also contains triterpenoids, sesquiterpene lactones, and flavonoids, and it has anti-inflammatory, antibacterial, anti-allergic, antitumor, and hepatoprotective effects [16,17,18,19,20,21,22]. According to recent studies, natural sesquiterpene lactone 6-O-angeloylplenolin isolated from CMX exerts anti-neuroinflammatory effects by regulating inflammatory cytokines and nuclear factor-κB (NF-κB) signaling pathways in murine microglial BV2 cells. Helenalin isolated from CMX inhibits hepatic stellate T6 cells and miR-200a-mediated phosphoinositide 3-kinase/Akt and NF-κB pathways in vivo, inhibiting hepatic stellate cell activation [23,24]. It is a Janus kinase/signal transducer and activator of transcription (JAK/STAT) inhibitor, and can be used to control overactivated JAK and STAT signaling [25]. According to preceding studies, brevilin A is a JAK/STAT inhibitor with potential therapeutic applications linked to antifibrotic and anticancer properties [26,27,28,29]. In addition, arnicolide and microhelenin C, including arnicolide C and arnicolide D, are sesquiterpene lactones found in CMX, and arnicolide D is known to be effective against nasopharyngeal carcinoma and triple-negative breast cancer cells [30,31,32,33]. Recently, CMX was found to upregulate the expression of growth factors such as vascular endothelial growth factor (*Vegfa*) and insulin-like growth factor 1 (*IGF1*) through the Wnt/β-catenin, extracellular signal-regulated kinase, and c-Jun N-terminal kinase signaling pathways in human hair follicle dermal papilla cells, indicating that CMX may effectively prevent hair loss [34]. Transforming growth factor beta (*Tgfb*) is a cytokine, and there are three types: *Tgfb1*, *Tgfb2*, and *Tgfb3*. It is a factor that inhibits the growth of hair follicle epithelial cells and induces cell degeneration, and *Tgfb1* and *Tgfb2* are known as a role of inducing hair loss [35]. 

Therefore, it is essential to develop a therapeutic agent that is effective, is easy to absorb, and has few or no side-effects. This study examined whether DN106212 promoted hair growth in C57BL/6 mice.

## 2. Materials and Methods

### 2.1. DN106212 Purified from Centipeda Minima Extraction

*C. minima* was purchased from a natural herbarium (Chungbuk Republic of Korea) in December 2019. One of the authors (J.P.) identified the material. A specimen of the material (CM-2019-001) was deposited in the herbarium of Kyungsung University. CMX was prepared by D. Nature Co. Ltd. (Seongnam, Republic of Korea) by inducing phase separation in the emulsion to efficiently separate brevilin A from *C. minima* [29]. CMX was extracted with ethanol and further fractionated with hexane. The fraction was concentrated (non-emulsion) using MCT oil (surfactant) to obtain only the upper layer for the preparation of DN106212.

### 2.2. HPLC-UV Conditions

To determine the amount of brevilin A, amicolide D, amicolide C, and microhelenin C compounds of CMX, 10 μL samples were injected into an HPLC ultraviolet (UV) system (Waters Alliance, e2695 Seperation module, Waters Corporation, Milford, MA, USA) with an autosampler and a UV detector. Chromatographic separation was carried out on a SUPERSIL column ODS-III (250 mm × 4.6 mm, 5.0 μm) and the UV wavelength was measured at 224 nm. The column oven temperature was 40 °C throughout, and the flow rate of the mobile phase was 1.0 mL/min. The mobile phase consisted of 0.1% formic acid and methanol (45:55).

### 2.3. Experimental Mice and Their Management

We used 6 week old female C57BL/6 mice purchased from DBL (Chungbuk, Republic of Korea) as the experimental animal. During the experiment, temperature (24–26 °C), humidity (55–60%), and a 12 h light cycle were maintained in the breeding room. After allowing mice to intake food and water freely and adapt to the environment for 7 days, we used them in the experiment. The study was approved by institutional animal care and use committees of Gachon University (GU1-2022-IA0041).

### 2.4. Hair Growth Evaluation

On days 0, 7, 10, 13, and 16, we took a photograph of the mice using a digital camera (Canon IXUS, Tokyo, Japan) under anesthesia with isoflurane. Photographic data were analyzed using Leica LAS Image Analysis (Leica Microsystems, Wetzlar, Germany), and the hair growth area was calculated as a percentage.

### 2.5. Histological Study

To observe hair growth, the back skin of the mice was cut using sterilized surgical scissors. Skin specimens were stored in 10% formalin at room temperature for 24 h for fixation and then embedded in paraffin using an auto processor (Excelsior ES, Massachusetts, Thermo Scientific, Waltham, MA, USA). The embedded tissue was sectioned into 5 μm sections, stained with hematoxylin and eosin (H&E, Sigma-Aldrich, St. Louis, MO, USA), and observed under an optical microscope at 40× magnification. 

### 2.6. Experimental Studies with DN106212

The experimental group was randomly assigned to the control, TF (positive control), and DN106212 groups, with six mice in each group. After the mice were anesthetized using the inhalation method using isoflurane, the back skin hair of the 6 week old mice was removed using a hair clipper. Afterward, we used Niclean cream (Ildong Pharmaceutical, Seoul, Republic of Korea) to remove all remaining fine hairs. After a recovery period of 24 h, the back skin was treated. We applied DMSO (negative control group), 2% TF (positive control group), or DN106212 in the same amount in a liquid state to half of the mouse back twice a day at a specific time. The treated skin was used for sample collection.

### 2.7. Quantitative Real-Time Polymerase Chain Reaction (qPCR) Analysis

Total RNA was extracted from the back skin of mice, which was cleaved using the method suggested by the manufacturer (QIAGEN, Valencia, CA, USA), and the RevertAid First-Strand cDNA Synthesis Kit (Thermo, Scientific, Waltham, MA, USA) was used to synthesize cDNA. We then mixed relative amounts of genes with PowerUpSYBR Green Master Mix and a series of primers and performed qPCR under the following conditions for 40 cycles: pre-denaturation at 95 °C for 30 s, denaturation at 95 °C for 5 s, annealing at 60 °C for 10 s, and extension at 72 °C for 15 s. We used the QuantStudio 3 Real-Time PCR System for qPCR and analyzed the expression level of *Actb* using the 2^−∆∆Cq^ method for relative quantification of each gene (Table 1).

### 2.8. Network Pharmacological Analyses

Network pharmacological analysis was performed by predicting targets and identifying pathways related to CMX. The predicted targets of CMX were obtained from the traditional Chinese medicine systems pharmacology database and analysis platform (TCMSP) (http://tcmspw.com/tcmsp.php, accessed on 23 June 2021), Bioinformatics Analysis Tool for Molecular mechanism of traditional Chinese medicine (BATMAN-TCM) (http://bionet.ncpsb.org/batman-tcm/, accessed on 23 June 2021), and traditional Chinese medicine mesh (TCM-mesh) (http://mesh.tcm.microbioinformatics.org/, accessed on 23 June 2021) [36]. The obtained interactions were experimentally validated or predicted using machine learning methods (support vector machine and random forest for TCMSP, similarity-based method for BATMAN-TCM, and random forest for TCM-mesh). The performance of these methods for the prediction of compound-target interactions has been shown to be reliable.

The pathways related to the targets were identified using gene set enrichment analysis (GSEA) using Enrichr (http://amp.pharm.mssm.edu/Enrichr/, accessed on 23 June 2021) [37]. EnrichR computes the *p*-value using Fisher’s exact test for the pathways of the gene list of interest (target genes). The adjusted *p*-value is calculated using the Benjamini–Hochberg method to correct for multiple hypothesis testing. The z-score is computed using a modification of Fisher’s exact test and assesses the deviation from the expected rank. Finally, the combined score is calculated using the adjusted *p*-value and z-score. The combined score is calculated as the logarithm of the multiplication of the *p*-value and z-score.

A compound–target network is a bipartite network in which nodes are defined as compounds or targets and the edges between compounds and targets are defined as compound–target interactions. The compound–target network of CMX was constructed and visualized on the basis of information retrieved from databases using Cytoscape [38]. The pathways associated with hair loss were obtained from a study that compared and analyzed gene expression profiles from the Gene Expression Omnibus databases [39].

### 2.9. Statistical Analysis

The experimental results are expressed as the mean ± standard deviation. Statistical analysis was performed using the GraphPad PRISM statistical package (ver 5.00, Graphpad software Inc., San Diego, CA, USA). Nonparametric comparisons of samples were conducted using the Kruskal–Wallis test to analyze the results. The difference was considered statistically significant at *p* < 0.05.

## 3. Results

### 3.1. HPLC Chromatograms of Standards and DN106212

**Figure 1:**
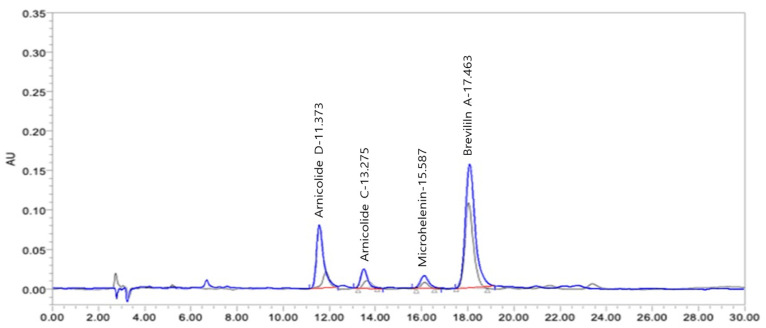
HPLC chromatograms of DN106212. Overlapped HPLC chromatogram of the compound DN106212 and the CMX fraction used in previous study (grey line) (Kim et al. 2021 [34]) and an enhanced purification (blue line).

**Figure 2:**
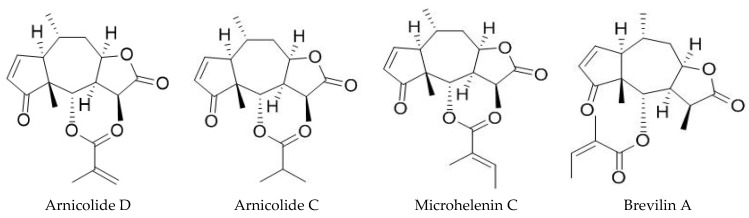
Chemical structures of arnicolide D, arnicolide C, microhelenin C, and brevilin A.

HPLC was used to determine the contents of arnicolide D, arnicolide C, microhelenin C, and brevilin A in DN106212, and their contents were compared with the standard. The standard manufacturer was Chengdu Biopurify Phytochemicals Ltd. (PRF9071704, 99.18% purity). The brevilin A standard was prepared at concentrations of 10, 25, 50, 100, and 250 ppm and used as a standard solution for the calibration curves. For the sample solution, 2 mL of DN106212 was used, and ethanol (10 mL) was added according to the marked line. The overlapped HPLC chromatograms of DN106212 and the CMX fraction used in this and a previous study are shown in Figure 1 [34]. Their chemical structures are shown in Figure 2. The retention times of the components of DN106212 were 11.373, 13.275, 15.587, and 17.463. By comparing the contents of arnicolide D, arnicolide C, microhelenin C, and brevilin A in STD and DN106212, the contents of icolide D, arnicolide C, microhelenin C, and brevilin A in DN106212 were confirmed to be 235.9, 91.8, 33.2, and 549.3 µg/mL, respectively. The content of brevilin A in DN106212 was approximately 1.5 times higher than that of the other three combined, and the chromatographic results are summarized in Table 2.

### 3.2. Effects of DN106212 on Hair Growth in Mice

**Figure 3:**
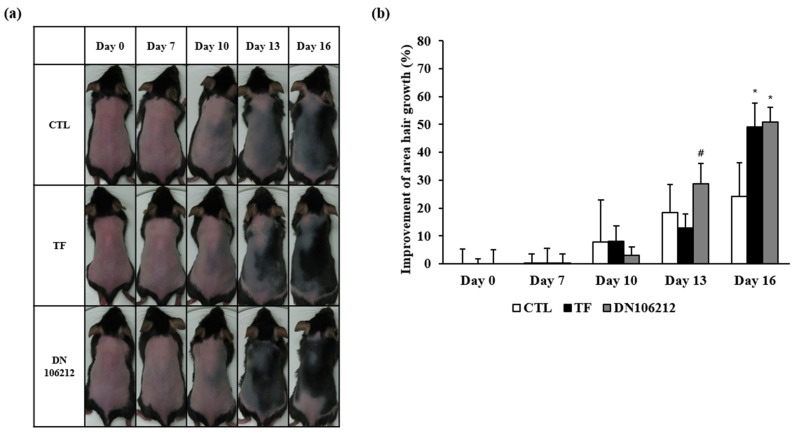
The effect of DN106212 on hair growth in C57BL/6 mice. (**a**) Indicated doses of DN106212 and TF were applied topically to the dorsal skin twice a day for 16 days. (**b**) Comparison of percentage of area hair regrowth on days 0, 7, 10, 13, and 16 among all groups. Data are presented as the mean ± SD, n = 6 in each group; * *p* < 0.05 (compared with CTL), ^#^ *p* < 0.05 (compared with TF).

The results of the visual observation of hair growth after taking a picture are shown in Figure 3a. Most C57BL/6 mice become dormant at 6–7 weeks of age, and their skin color becomes pink. This study observed the hair growth effect of DN106212 through a comparative experiment with the positive control TF, which is known to be effective for hair growth. All mice showed pink skin after depilation, and the same gray color appeared on day 10. From the 13th day, the TF and DN106212 groups showed hair growth earlier than the control group. From the 16th day, DN106212 group showed faster hair growth than the TF group. The results of the analyses using LAS software to quantify hair growth are shown in Figure 3b, where the skin appearance—pink, gray, or hairy—was measured by dividing it by light and dark according to the degree of hair growth using LAS software. The measured area was converted into the ratio of the total area to be depilated and averaged, and the hair growth area was expressed as a percentage. Until the seventh day, there were no significant differences among the groups; however, on the 10th day, the CTL group (7.87% ± 15.04%) and the TF group (8.15% ± 5.47%) showed hair growth. On the 13th day, the CTL group showed a growth rate of 18.43% ± 10.09%, the TF group showed a growth rate of 12.77% ± 5.16%, while the DN106212 group showed a significantly higher growth rate of 28.63% ± 7.39%. On the 16th day, the TF group showed a significant increase in hair growth rate at 49.16% ± 8.52% compared to the CTL group (24.14% ± 12.16%), and the DN106212 group also increased significantly at 51.00% ± 5.28% (see Supplementary Table S1). As a result, the TF group and the DN106212 group showed the highest hair growth effect, demonstrating that DN106212, like TF, has a beneficial effect on hair growth. 

### 3.3. Effect of DN106212 on Hair Follicles in Mice

**Figure 4:**
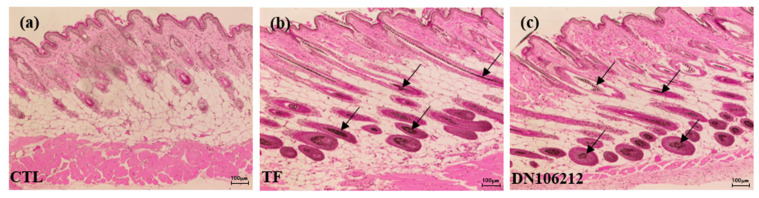
Results of 16 day application of topical control (**a**), TF (**b**), and DN106212 (**c**). Different hair growth patterns can be observed. The arrow indicates that the hair follicle is generated in the deep dermis. Longitudinal sections of the dorsal skin (16 day) were stained using H&E and viewed under 40× magnification. Scale bar: 100 µm. Each image presented is a representative of six mice.

The results of the histological analysis of H&E staining and collection of skin tissue from the mice are shown in Figure 4. The control group showed small hair follicles, but no mature hair follicles were observed. The number and size of hair follicles increased in the subcutaneous layer in the TF group. In the DN106212 group, more mature hair follicles were found in the dermis and epidermis compared with the TF group. The DN106212 treatment group showed no inflammatory or other pathological features; however, the size of the mature hair follicles grown in the dermis and subcutaneous layer was large, and the shape of the hair follicle was formed as some hair follicles penetrated the skin and hair growth began.

### 3.4. Effects of DN106212 on Vegfa, Igf1, and Tgfb1 in Dorsal Skin of Mice

**Figure 5:**
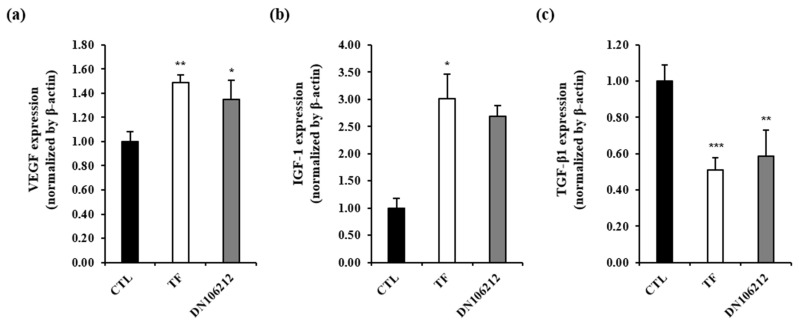
Comparison of the expression of *Vegfa*, *Igf1*, and *Tgfb1* mRNA evaluated with RT-PCR. The relative expression levels of (**a**) *Vegfa*, (**b**) *Igf1*, and (**c**) *Tgfb1* were determined using qPCR analysis. * *p* < 0.05, ** *p* < 0.01, and *** *p* < 0.001 compared with the control group.

The results of the gene expression analysis of the hair growth effect of DN106212 extracted from CMX in a hair growth animal model using real-time PCR are shown in Figure 5. The gene expression levels of *Vegfa* and *Igf1*, which are known regulators and promoters of hair growth, were compared between the control group and the sample group. The expression levels of both genes increased as follows: *Vegfa* 1.49 ± 0.07 and *Igf1* 3.01 ± 0.45 in the TF group; *Vegfa* 1.35 ± 0.16 and *Igf1* 2.68 ± 0.51 in DN106212 group. The expression of *Tgfb1*, which is a hair loss-inducing factor, was significantly reduced to 0.51 ± 0.07 in the TF group and 0.58 ± 0.14 in the DN106212 group compared with the control group.

### 3.5. Network Pharmacology Analysis

We conducted network pharmacological analysis to investigate the underlying mechanisms of CMX. We identified 40 target genes of CMX from three network pharmacology databases: TCMSP, BATMAN-TCM, and TCM-mesh [36,40,41]. These targets were either experimentally validated or predicted using machine learning algorithms. To test whether these targets were significantly associated with pathways related to hair loss, GSEA was performed according to the KEGG database [42]. Among the 17 pathways associated with hair loss, we found that the CMX targets were significantly enriched in eight pathways with high combined scores (Table 3). This suggests that the effects of CMX on the modulation of hair loss are mediated by these pathways.

**Figure 6:**
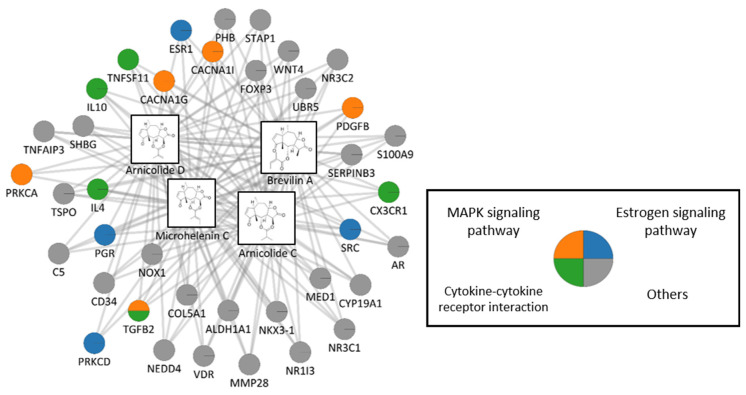
Compound–target network of CMX. Boxes and circles represent the compounds and targets, respectively. Edges between compounds and targets indicate the interactions between them. The targets of CMX were colored to indicate three pathways with a high combined score among pathways related to hair loss. CMX, emulsion extract from *Centipeda minima*.

To elucidate the compound–target interactions, we constructed and visualized the compound–target network between CMX and its target genes (Figure 6). There were five, five, and four related targets for the estrogen signaling pathway, MAPK signaling pathway, and cytokine–cytokine receptor interaction pathway, respectively. We also found that all CMX compounds interacted with TGFB2, which is involved in the MAPK signaling pathway, cytokine–cytokine receptor interaction, and osteoclast differentiation pathway. Considering the association between the TGF-β family and male hair loss, our analysis suggests that the effects of CMX on hair loss could be exerted via regulation of various hair loss-related proteins, including TGF-β, which underlies the therapeutic potential of CMX in treating hair loss [43].

## 4. Discussion

We evaluated whether DN106212 has a better hair growth-promoting effect than 2% TF in hair-removed C57BL/6 mice. The effect of TF treatment was consistent with a previously proposed TF hair growth effect [44]. When treated with TF and DN106212 at regular time intervals, DN106212 showed a better hair growth effect than TF on the 16th day. Moreover, *Vegfa*, *Igf1*, and *Tgfb1* expression were significantly modulated in the DN106212-treated group, indicating that DN106212 has potential as a therapeutic agent for hair loss. Network pharmacology analysis suggested potential targets and pathways through which CMX acts on hair loss, including the TGF-beta family. In conclusion, our findings highlight the potential of DN106212 as a promising therapeutic agent for hair loss, while also presenting a strategy for elucidating the systems-level mechanisms of natural products in promoting hair growth.

Since skin pigmentation in C57BL/6 mice develops only during the growth period, it is a useful model for distinguishing the presence or absence of hair growth-promoting effects. Testosterone in male mice can promote the growth of fur and hair, which can affect the results [45,46]. As such, we chose a female mouse model, as in previous studies, in order to control for sex-specific variables such as body weight, and to ensure consistent experiments [47,48,49]. The TF and DN106212 groups showed a clear difference in hair growth from the control treatment group from the 13th day, and, on the 16th day, it was confirmed that the DN106212 treatment group had a higher hair growth rate than the TF treatment group. The results of the study are similar to those reported in clinical studies that CMX and brevilin A promote hair regrowth as JAK3 inhibitors in patients with hair loss [50]. This means that DN106212 has a positive effect on the rate of hair growth and a strong effect on the early induction of the growth phase. The group treated with TF and DN106212 for 16 days showed a positive effect on hair growth compared to the CTL group. According to a preceding study, when 2% TF and 5% minoxidil, well known as hair loss treatment agents, were topically applied on mice, the number of hair follicles increased markedly in histopathology and revealed that TF was more effective in promoting hair growth [51]. This study monitored the growth and size of hair follicles, which are important indicators of hair growth promotion, by staining the skin and visualizing it under a microscope. These findings indicate that DN106212 is effective in promoting hair growth as it stimulates the hair growth phase.

This study also examined the mRNA expression levels to elucidate the mechanisms of hair growth factors and inhibitory factors in mouse skin tissues. We confirmed that the expression ratio of *Vegfa* and *Igf1* growth factors increased significantly in the DN106212 treatment group compared with that in the control group, and the expression ratio of *Tgfb1*, a growth-inhibitory factor, decreased significantly in the DN106212 treatment group compared with that in the control group. The results of this study are consistent with the findings that the expression of *Vegfa*, *Igf1*, and *Tgfb1* in mice is closely related to hair growth [52]. Hair loss caused by scar-forming lupus, folliculitis, burns, and injury cannot be resolved because the hair follicles are destroyed [53]. In contrast, scar-free hair loss, such as hereditary androgenic hair loss, partial alopecia, and telogen alopecia, may be recovered if the hair follicle is maintained and the symptoms are relieved [54]. Male, female, and partial alopecia are mainly caused by male hormones [55]. Testosterone, a male hormone, is converted into DHT, which causes hair loss by interacting with 5α reductase around the hair root [56]. DHT combines with androgen receptors in the hair paper cell membrane, inhibiting the synthesis of *Igf1*, fibroblast growth factors, and *Vegfa* involved in hair follicle regeneration, weakening hair, and promoting hair loss through apoptosis. As a result, the apoptosis factors bone morphogenic protein dickkopf-1 and *Tgfb1* are generated, inhibiting the division of hair root cells, making the hair follicle smaller, and pushing the hair follicles in the degenerative phase [57]. In the previous study, rats treated with DHT showed a decrease in the expression of *Igf1*, leading to a reduction in the number of proliferative cells in hair follicles and a decrease in hair growth [58]. Therefore, it is assumed that DHT can suppress hair growth in mice by inhibiting *Igf1* production in the dermal papilla through interaction with androgen receptors, which in turn suppresses sensory neuron stimulation in the hair follicles [59]. In this experiment, treatment with DN106212 resulted in an increase in the expression of *Igf1*. Therefore, apoptotic factors should be reduced, or growth factors should be activated.

As a hair loss treatment, minoxidil and finasteride are well known for percutaneous application and oral use [60,61]. However, side-effects, such as decreased sexual desire, itchiness, and red spots, have been reported for the drugs [62,63]. The use of finasteride is also limited to women because it can interfere with the estrogen/testosterone balance, leading to potential risk of estrogen-mediated malignant. Tofacitinib (TF) is a synthetic molecule approved by the Food and Drug Administration for the treatment of rheumatoid arthritis and is recommended for adult patients suffering from the condition [64]. It mainly inhibits enzyme activity, including that of JAK1 and JAK3 [65]. Topically applied TF might facilitate hair growth by regulating alopecia areata [66]. However, TF fails to reliably promote hair growth. It is impossible to take immunosuppressants, such as JAK inhibitors, throughout one’s lifetime; furthermore, hair loss may recur or become more severe if the medication is stopped [67].

Therefore, it is necessary to develop a hair loss treatment agent made of natural materials that is effective for hair growth and has few side-effects. This study confirmed that DN106212 effectively promoted hair growth by regulating the gene expression levels of *Vegfa*, *Igf1*, and *Tgfb1* in mice. However, further studies should be conducted on the effects and side-effects by increasing the sample size and duration of application, as well as investigating the long-term effects of DN106212 on hair growth. Nevertheless, in this study, DN106212 showed a better hair growth-promoting effect than TF, highlighting its promise as a new therapeutic for hair loss.

## Figures and Tables

**Table 1:** List of primers for real-time PCR of the DN106212.

Genes	Sense (5′→3′)	Antisense (5′→3′)
*Vegfa*	CTGGATATGTTTGACTGCTGTGGA	CTGGATATGTTTGACTGCTGTGGA
*Igf1*	CACTGACATGCCCAAGACTCAGA	TCCGAGTTGCCTCCGTTACC
*Tgfb1*	CTC CCG TGG CTT CTA GTG C	GCC TTA GTT TGG ACA GGA TCT G
*Actb*	TTCCAGCCTTCCTTCTTG	GGAGCCAGAGCAGTAATC

**Table 2:** Contents of arnicolide D, arnicolide C, microhelenin C, and brevilin A in DN106212.

	Arnicolide D	Arnicolide C	Microhelenin C	Brevilin A
Content (µg/mL)	235.9	91.8	33.2	549.3

**Table 3:** Enrichment analysis of CMX target-associated pathways related to hair loss.

Term	Overlap	Adjusted *p*-Value	Combined Score	Genes
Estrogen signaling pathway	4/137	0.002401	156.9739	*SRC;PRKCD;PGR;ESR1*
MAPK signaling pathway	5/295	0.003058	86.22343	*CACNA1I;TGFB2;PDGFB;PRKCA;CACNA1G*
Cytokine–cytokine receptor interaction	5/294	0.003058	86.68829	*IL10;IL4;CX3CR1;TGFB2;TNFSF11*
Osteoclast differentiation	3/127	0.010617	86.71594	*TGFB2;TNFSF11;NOX1*
Chemokine signaling pathway	3/190	0.020279	47.03707	*CX3CR1;SRC;PRKCD*
Focal adhesion	3/199	0.022327	43.75158	*SRC;PDGFB;PRKCA*
Rap1 signaling pathway	3/206	0.02344	41.43427	*SRC;PDGFB;PRKCA*
Fc epsilon RI signaling pathway	2/68	0.023807	82.16884	*IL4;PRKCA*

## Data Availability

Data is contained within the article.

## Supplementary Materials

The following supporting information can be downloaded at: https://www.mdpi.com/article/10.3390/cimb45060322/s1, Figure S1: Skin testing of DN106212 at various concentrations in C57BL/6 mice (n = 6); Table S1: The effect of DN106212 on hair growth in C57BL/6 mice.
