# Supplementary material for: Hair Growth Effect of DN106212 in C57BL/6 Mouse and Its Network Pharmacological Mechanism of Action
Source: Curr Issues Mol Biol. 2023 Jun 9;45(6):5071–83. doi: 10.3390/cimb45060322 (PMC10297088; doi:10.3390/cimb45060322)
Supplement: Supplementary file 1 [file cimb-45-00322-s001.zip › cimb-2367067-supplementary.pdf]

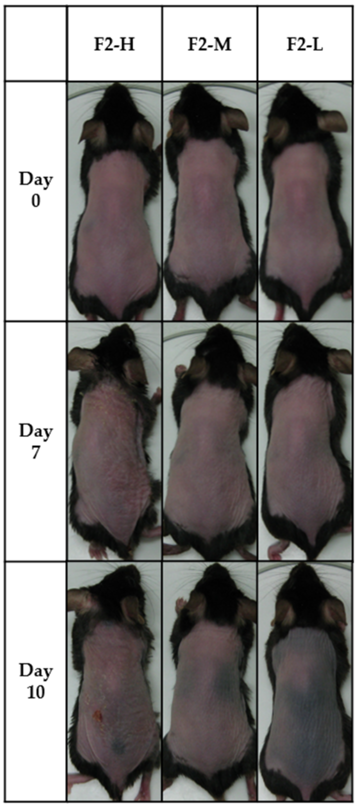

Figure S1. Skin testing of DN106212 at various concentrations in C57BL/6 mice (n=6). DN106212 was topically applied to the mouse skin at three different concentrations, with each concentration receiving a 100 μL treatment for a duration of 10 days. F2-H; high dose, F2-M; medium dose, F2-L; low dose.

Table S1. The effect of DN106212 on hair growth in C57BL/6 mice. Comparison of percentage of area hair regrowth on day 0, 7, 10, 13, and 16 among all groups. Data were presented as mean ± SD, n=6 in each group. **p*<0.05 (compared with CTL) and #*p*<0.05 (compared with TF).

(%)	CTL	TF	DN106212
**Day 0**	0.00 ± 5.19	0.00 ± 1.77	0.00 ± 5.12
**Day 7**	0.02 ± 3.43	0.00 ± 5.55	0.00 ± 3.42
**Day 10**	7.87 ± 15.04	8.15 ± 5.47	2.94 ± 3.14
**Day 13**	18.43 ± 10.09	12.77 ± 5.16	28.63 ± 7.39#
**Day 16**	24.14 ± 12.16	49..16 ± 8.52*	51.00 ± 5.28*
